# Construction and validation of CAPSES scale as a composite indicator of SES for health research: an application to modeling social determinants of cardiovascular diseases

**DOI:** 10.1186/s12889-023-15206-9

**Published:** 2023-02-09

**Authors:** Mohsen Asadi-Lari, Reza Majdzadeh, Mohammad Ali Mansournia, Saharnaz Nedjat, Kazem Mohammad, Bahman Cheraghian

**Affiliations:** 1 Oncopathology Research Centre, University of Medical Sciences, Tehran, Iran; 2 School of Health and Social Care, University of Essex Colchester, Colchester, UK; 3 Department of Epidemiology and Biostatistics, School of Public Health, Tehran University of Medical Sciences, Tehran, Iran; 4 School of Public Health, Tehran University of Medical Sciences, Tehran, Iran; 5 Department of Biostatistics and Epidemiology, School of Public Health, Ahvaz Jundishapur University of Medical Sciences, Ahvaz, Iran

**Keywords:** CAPSES scale, SES, Social determinants, Cardiovascular diseases, Urban HEART, Iran

## Abstract

**Background:**

The main objective of this study was to construct and validate a composite socioeconomic status indicator containing material capital, human capital, and social capital (CAPSES scale) and also appropriate it for CVDs in a large population-based study.

**Methods:**

This cross-sectional study, the Urban HEART-2 project, was conducted in Tehran, Iran, in 2011. A total of 34,116 households covering 118,542 individuals were assessed in this study. A 14-parts questionnaire was completed for all selected households. All the gathered data were based on the participants’ self-reports. Literacy, wealth index, expenditure, skill level, and Townsend index were used as SES indexes. CVDs, including Hypertension, Myocardial infarction, and stroke, were considered the main outcomes. A structural equation model (SEM) was used to construct a CAPSES scale and a composition index of SES. Criterion validity and Construct validity were used to assess this scale.

**Results:**

A total of 91,830 subjects consisting of 33,884 (49%) men were included in this analysis. The mean age of the participants was 41.5 ± 11.37 years. Among the assessed participants, 5904(6.4%) reported hypertension, 1507(1.6%) myocardial infarction, and 407(0.4%) strokes. The overall weighted prevalence of self-reported cardiovascular events (hypertension, stroke, and MI) was 8.03% (95%CI: 7.8–8.2). Inverse associations were seen between the CAPSES scale and its domains with CVDs, adjusted for sex, age, BMI, smoking, and diabetes by a multiple logistic regression model.

**Conclusion:**

The CAPSES scale was significantly associated with stroke and hypertension. Our findings showed that the CAPSES index could be useful for public health research.

**Supplementary Information:**

The online version contains supplementary material available at 10.1186/s12889-023-15206-9.

## Background

Social determinants of health (SDH) encompass the economic, social, environmental, and psychosocial factors influencing health [1]. These conditions are income, wealth, education, occupation, working conditions, job security, housing, health care services, culture, religion, and social safety nets.SDHs are increasingly being addressed as causal factors for health disparities [2].

Social determinants have been recognized to be a significant factor in the development of cardiovascular diseases (CVDs) and related risk factors [3, 4].

Cardiovascular diseases (CVDs) remain the number one cause of death worldwide and are responsible for an estimated 17.9 million deaths each year (31% of all deaths globally) [5]. Cardiovascular diseases are the leading cause of mortality [6, 7] and contributors to the global burden of disease [8]. Harper et al. [9] implied that population levels of CVDs depend on the prevalence of major risk factors, and levels of risk factors depend on macro-social forces and socioeconomic position. SDH plays a significant role in the development of cardiovascular disease (CVD) risk factors and CVD morbidity and mortality [1]. Accumulating evidence suggests that there is a socioeconomic status (SES) disparity in CVDs: individuals with lower SES tend to have worse CVDs risk profiles and outcomes [10–12], and several CVDs risk factors, including hypertension, diabetes, and obesity [13–15]. The effects of SES on CVDs may be mediated through several biological, behavioral, and social risk factors [10, 16].

SES is commonly documented as complex and multidimensional, integrating different components that may be either material, social, or both [17]. The new CAPSES scale is a composite indicator that includes more social complexities in the socioeconomic status compared to traditional composite indicators, which Oakes and Rossi proposed in 2003 [18]. In the calculation of the CAPSES scale, social capital is considered one of the dimensions of socioeconomic status, which is one of the distinguishing features of this index compared to traditional composite indicators [19].

The main objective of this study was to determine the validity of a new alternative SES indicator containing material, human and social capital (CAPSES scale) and appropriateness for CVDs in a population-based study in Iran. Also, we intend to provide suitable solutions for preventing and controlling these diseases by identifying and introducing the most relevant socioeconomic factors to cardiovascular diseases.

## Methods

### The aim, design, and setting of the study

The second Urban Health Equity Assessment and Response Tool (Urban HEART-2) project was a large population-based and cross-sectional study conducted in Tehran, the capital of Iran, from 23 September to 22 October 2011. A total of 118,542 participants were interviewed at their houses by qualified interviewers trained during a two-day workshop [20]. All methods of conducting and data gathering have been done according to the Urban HEART-2 guideline and have been approved by the Iran University of Medical Sciences. The Urban HEART project is well-designed, a pilot study was conducted before the main study, and the interviewers were well-trained. As a result, the quality of completing the questionnaires was favorable. In the quality control by supervisors, the interviewers contacted the participants again to complete the missing data.

### Sampling method and sample size

A multistage cluster random sampling method was used in the study. The sample size based on the Cochrane formula was calculated. The total sample size was 34,116 households covering 118,542 individuals. Because some socioeconomic indicators, including skill level, did not make sense for people under 20 years; as a result, these people were excluded. Finally, the analysis was performed on the remaining 91,830 individuals' data after excluding participants under 20 years (*n* = 26,712). The details of this study's sample size determination and sampling method have already been completely published [21].

### Measurements

There were 3 types of questionnaires consisting of 20 parts. The first volume (pink color) contained 14 parts, including household information, demographic information of all household members, socioeconomic information, diseases (e.g., hypertension, cardiovascular, stroke, and myocardial infarction), etc. The second volume (blue color) contained 6 parts, which included individual information in various fields, including mental health, social capital, etc., and based on an age-sex table; only one selected person completed it from each household. Finally, the third questionnaire (green color), which contained many questions in the field of household nutrition, randomly selected one of the households from each block of eight households was questioned. All the gathered data were based on the participants’ self-reports. For any participant, the three main forms of cardiovascular diseases include heart attack, stroke, and hypertension, which a physician diagnosed at the time of the survey. The validity and reliability of GHQ-28 [22] and social capital [23] have been confirmed in previous studies on the Iranian population.

### Health status in general question

All the participants were asked, "How do they classify their health status in general?". This question had five options "very good, good, average, bad, and very bad". We divided the people who chose the first two options into the healthy group and those who chose the next three options into the sick group.

### GHQ-28 questionnaire

The general health questionnaire consisted of 28 four-choice questions, the range of answers of which was "much less than usual, less than usual, like usual, and more than usual". There are two scoring methods for general health questionnaires. In this research, the Likert scoring method was used so that the minimum score of the questionnaire was 0 and the maximum score was 84, where a higher score indicates a worse public health condition. In this questionnaire, a cut-off point of 23 was used in such a way that scores lower than it was included in the healthy and equal group and more than that were included in the group with psychological disorders.

### Expenditure per capita (EPC)

In this study, we used the total family expenditure per month to calculate the expenditure per capita for everyone.

### Income adequacy

To measure income adequacy, we used a question with a three-point Likert scale was used: "not at all sufficient", "somewhat sufficient," and "completely sufficient".

### Skill level

We used the International Standard Classification of Occupations 2008 (ISCO-08) for job classification. There is a four-level hierarchically structured classification in the ISCO-08. This classification ranges from Skill level I, which involves the performance of the simplest and manual tasks, to skill level IV, which involves the performance of the most complex tasks that require complex problem-solving, decision-making, and creativity [24].

Including landline phone, mobile phone, bathroom, kitchen, toilet, car, motorcycle, freezer, microwave, computer, and dishwasher, as well as the per capita of the building (less than the median—equal to and greater than the median), the per capita of the room (per person) less than one room—one room and more), the type of residence (owned-rented) were all included in the analysis as a dichotomous variable.

### Wealth index

It was calculated using principal component analysis (PCA) on 14 assets consisting of owning a fridge, a personal computer, a telephone, a mobile phone, a washing machine, a microwave oven, a car, a motorcycle, a kitchen, a bathroom, a toilet, house ownership, number of rooms per capita (less than one vs. one and more), and area of the house (below the median vs. above the median). In principle component analysis, the first component explains the largest proportion of the total variance; thus, assets more unequally distributed across the sample had a higher weight in the first component. The weights (coefficients) for each asset from this first component were used to generate the wealth scores, with a higher score indicating higher wealth status and vice versa [25].

### Townsend Index

Townsend Index, as an area-level index of socioeconomic status, is a composite index based on four variables: the proportion of unemployment, the proportion of households with no car, the proportion of households that are not owner-occupied, and the proportion of households with overcrowding (more than one person per room). To calculate the index, we obtained a sum of each variable's standardized scores (z scores) to obtain an overall score. A greater score indicates higher levels of deprivation [26].

### Self-rated health

Self-rated health was measured by a question asking if the participant would rate their health as very good, good, moderate, fair, or poor. We divided people into healthy and disease groups, so those who chose the excellent and very good were healthy, and those who chose the next three options were divided into the disease group.

### Capital indicators

#### 1- Material capital

Urban Heart questionnaire collection of 14 assets. We extracted, using PCA, five assets (fridge, washing machine, microwave oven, computer, and car) from 14 assets used in the model as material domain indicators.

#### 2- Human capital

To assess this domain, we used four indicators of literacy and skill level, and if the person is not a householder, the job and education of the father as the householder.

#### 3- Social capital

The social capital questionnaire was designed to measure which has 56 questions in 6 domains consisting of collective activities (9 questions), voluntary activities (9 questions), social networks (10 questions), social belonging (9 questions), trust (9 questions) and reciprocal action (10 questions) were determined. Each question was on a 5-point Likert scale ranging from 1 = not at all to 5 = completely.

### Constructing the CAPSES

**Fig. 1:**
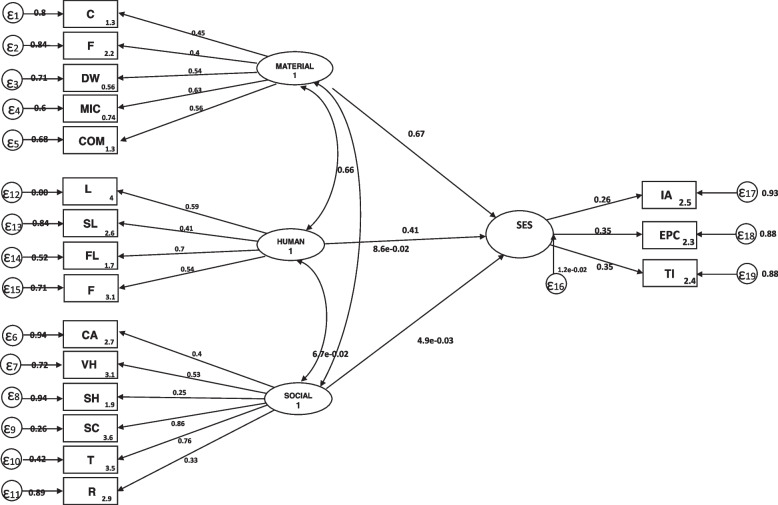
Structural equation model to estimate SES as a latent variable. C: Car; F: Frizer; DW: Dishwasher; MIC: Microwave; Com: Computer; L: Literacy; Skill level; FL: Father Literacy; FJ: Father Job; CA: Collective Activity; VH: Voluntary Help; SN: Social Network; SC: Social Coherence; T: Trust; R: Reciprocity

For constructing a composite indicator of SES, we used Material capital, Human capital, and social capital as capital dimensions that affect the person's SES. In our model, income adequacy (IA), expenditure per capita (EPC), and Townsend index (TI) were the indicator variables of SES. In general, the lower the CAPS score, the lower the socioeconomic level. Figure 1 shows a structural equation model (SEM) to estimate SES.

In the obtained SEM model, the coefficients of material capital and human capital domains were statistically significant from the three dimensions of socioeconomic status. In contrast, while small, the social capital coefficient was not statistically significant. Of these, the highest coefficient belonged to material capital with 0.67 ± 0.068, the human capital coefficient was 0.41 ± 0.064, and the lowest was social capital with 0.005 ± 0.035. The structural equation model of the relation between SES and capital dimensions is as follows:

Socioeconomic status = 0.67 (Material capital) + 0.41 (Human capital) + 0.005 (Social capital). We generated a CAPSES score from factor weights for each individual. A higher CAPSES score indicates a better socioeconomic status.

### Statistical analysis

PCA was used to construct the wealth index. Chi-square and odds ratio (OR) were used to assess univariate analysis associations. We used the structural equation modeling (SEM) method, and a special type called the Multiple Indicators Multiple Causes (MIMIC) models to develop the CAPSES composite indicator and determine the coefficients and weighting of the items of the CAPSES scale. In the MIMIC model ξ(Ksi): latent and independent variable; x: an obvious variable and dependent on the kai variable;(Delta) δ: error related to x variables;(Eta) η: hidden and dependent variable; y: obvious variable and dependent on the eta variable;(Epsilon) ε: error related to y variables; (Landa) λ: the relationship coefficient between the hidden variables (independent or dependent) and the obvious variables dependent on them, i.e., x or y; (Gamma) γ: the relationship coefficient between independent and dependent hidden variables; (Zeta) ζ: The error in the hidden and dependent variable, i.e., eta; (Beta) β: the relationship coefficient between hidden variables dependent on each other; (Teta) Ф: coefficient of relationship between independent hidden variables with each other. Three indices (Root mean squared error of approximation), RMSEA Chi-square, and Akaike's information criterion (AIC) were used to check the goodness of fit of structural equation models. The significance of the coefficients was reported with a p-value of less than 5%. We generated a CAPSES score from factor weights for each individual. In the Urban HEART-2 project, the missing percentage was low (below 1%). In dealing with missing data, we used the pairwise deletion method, which used all available data. In univariate regression, we included variables that p-value was less than 0.25 in the multiple regression model. In multivariate analysis, ORs from the Logistic Regression model were used to measure the association between the study variables. A *p* < 0.05 was considered statistically significant. STATA 12.0 software was used for all the statistical calculations.

## Results

A total of 91,830 subjects consisting of 33,884 (49%) men and 35,289 (51%) women were included in this analysis. The mean ± standard deviation (SD) age of the participants was 41.5 ± 11.37 years. The demographic and Socioeconomic factors of the study are presented in Table 1. Among the assessed participants, 5904(6.4%) reported hypertension, 1507(1.6%) myocardial infarction, and 407(0.4%) strokes. The overall weighted prevalence of self-reported cardiovascular events (hypertension, stroke, and MI) was 8.03% (95%CI: 7.8–8.2).

**Table 1:** Frequency distribution of Demographic and Socioeconomic factors

**Variable**		**Number**	**Percent**
**Age(years)**	20–29	25,498	27.8
	30–39	19,280	21.0
	40–49	17,167	18.7
	50–59	14,816	16.1
	60–69	8848	9.6
	70–79	4666	5.1
	80–89	1479	1.6
	+ 90	76	.1
**Sex**	Male	45,990	50.1
	Female	45,824	49.9
**Marital status**	Married	59,659	66.2
	Widow or Divorced	5917	6.6
	Single	24,599	27.3
**Literacy**	Illiterate	6684	7.3
	Primary school	8171	9.0
	Middle school	10,867	11.9
	High school	37,633	41.2
	University	27,914	30.6
**Skill level**	Level 1	2662	12.6
	Level 2	12,170	57.5
	Level 3	2591	12.3
	Level 4	3724	17.6
**Expenditure**	Lowest	17,709	19.6
	Low	17,823	19.8
	Moderate	17,851	19.8
	High	18,013	20.0
	Highest	18,835	20.9
**Wealth Index**	poorest	6364	20.3
	poor	6160	19.6
	moderate	6445	20.5
	rich	6315	20.1
	richest	6088	19.4
**Townsend Index**	Most affluent	20,006	22.9
	Affluent	20,734	23.8
	Moderate	17,325	19.9
	Deprived	12,040	13.8
	Most deprived	17,097	19.6

We construct Wealth Index using principal component analysis (PCA) on 14 assets. Among the assets, having a microwave oven, washing machine, and personal computer had the highest Factor scores (Table 2).

**Table 2:** factor loading calculated using principal component analysis (PCA) on 14 assets

Variable	Mean ± SD	Factor score
Telephone	0.965 ± 0.183	0.436
Mobile phone	0.917 ± 0.277	0.263
Personal computer	0.619 ± 0.486	0.559
Fridge	0.828 ± 0.378	0.518
Washing machine	0.207 ± 0.405	0.563
Microwave oven	0.301 ± 0.463	0.616
Car	0.633 ± 0.482	0.525
Motorcycle	0.191 ± 0.393	-0.084
Bathroom	0.998 ± 0.047	0.186
Kitchen	0.995 ± 0.071	0.370
Toilet	0.982 ± 0.133	0.411
House ownership	0.655 ± 0.472	0.300
Number of rooms per capita (less than one vs. one and more)	0.291 ± 0.452	0.281
Area of the house (below the median vs. above the median)	0.460 ± 0.498	0.473

### Validity of CAPSES

To assess the validity of the composite socioeconomic indicator CAPSES, two types of validity, i.e., criterion validity and construct validity, were used, the results of which are as follows.

### a.Criterion validity

For evaluating the criterion validity of the CAPSES score, the correlation coefficients of CAPSES with selected indicators to measure socioeconomic status was examined using partial correlation analysis. All coefficients were adjusted for age and gender factors. Table 3 presents the correlation coefficients among selected SES Measures.

**Table 3:** Pairwise Partial correlation coefficient among selected SES Measures as criterion validity

	**Literacy**	**Skill level**	**Expenditure**	**Wealth Index**	**Townsend index**	**CAPSES**
Literacy	1					
Skill level	0.33	1				
Expenditure	0.14	0.1	1			
Wealth Index	0.32	0.21	0.16	1		
Townsend index	-0.22	-0.15	-0.17	-0 .27	1	
CAPSES	0.57	0.29	0.21	0.78	-0.37	1

^*^All of the p-values are statistically significant (*p* < 0.001)

As seen in Table 3, all correlation coefficients obtained were statistically significant (*p* < 0.05), but in all except one case, the examined correlations are not strong and are often weak or moderate. However, the correlation magnitudes of the combined scale of CAPSES compared to other socioeconomic indicators were higher. Moreover, the highest correlation of CAPSES was with the wealth index (0.78) and then with literacy (0.57). On the other hand, the combined scale of CAPSES had the weakest correlation with household expenditure (0.21). Also, as expected, all the coefficients of the deprivation index were negative, representing this indicator's reverse correlation with other studied socioeconomic status indicators.

Considering that the correlation values ​​of each of these economic indicators are different from the other 5 indicators, to determine the mean strength of the correlation of each socioeconomic indicator with other indicators, mean magnitudes of correlation, regardless of their signs, were calculated and based on them, the rank of each indicator in terms of the strength of correlation with other socioeconomic indicators was determined. The CAPSES indicator, with step, had the highest mean correlation, and family expenditure, with 0.16, had the lowest mean correlation with other socioeconomic indicators (Supl a).

### b.Construct validity

The next objective was to assess the construct validity of CAPSES in terms of health outcomes. As seen in Table 4, for the general health questionnaire (GHQ-28) and the single item of self-rated health, the obtained odd ratios for all socioeconomic indicators except expenditure and Townsend index are less than one and significant. A high socioeconomic status score is related to a better general health condition (low score on GHQ). Among these, the combined CAPSES index compared with other socioeconomic indices had a stronger relationship with both single item of general health and the general health questionnaire so that for a one-unit increase in CAPSES index, the odds of health increased by 17.5% for the general health questionnaire and by around 43% for the single item of self-rated health.

**Table 4:** Adjusted odds Ratios of five SES Measures Predicting "Good Health" according to self-reported health and GHQ-28 questionnaire and CVD

SES indicator	Self-related health		GHQ questionnaire		Cardiovascular disease	
	OR (95% CI)	*p*-value	OR (95% CI)	*p*-value	OR (95% CI)	*p*-value
**Literacy**	0.83 (0.82–0.85)	< 0.001	0.90 (0.88–092)	< 0.001	0.95(0.93–0.96)	< 0.001
**Skill level**	0.76 (0.72–0.81)	< 0.001	0.92 (0.86–0.98)	0.008	1.03(0.96–1.10)	0.38
**Expenditure**	1.00 (0.99–1.001)	0.9	1.022 (1.014–1.031)	< 0.001	1.03(1.02–1.04)	< 0.001
**Wealth Index**	0.78 (0.76–0.80)	< 0.001	0.88 (0.86–0.90)	< 0.001	0.97(0.94–0.99)	0.007
**Townsend index**	1.08 (1.06–1.09)	< 0.001	1.082 (1.066–1.098)		1.03(1.01–1.05)	0.001
**CAPSES**	0.70 (0.68–0.72)	< 0.001	0.85 (0.82–0.87)	< 0.001	0.93(0.89–0.98)	0.007

The Odds Ratios have adjusted for age and sex

### Use of CAPSES in public health research

In our study, the relationship between CAPSES and cardiovascular diseases was investigated. Table 5 shows the negative association of CAPSES and its domain with Hypertension, Myocardial infarction, stroke, and overall CVDs, adjusted for sex, age, BMI, smoking, and diabetes based on logistic regression results. Among all cardiovascular diseases, CAPSES had the strongest relationship with stroke, so with a one-unit increase in CAPSES, the odds ratio of stroke decreased by around 15%. This magnitude was estimated to be 8.7% for hypertension and 7.5% for all cardiovascular diseases. However, this relationship was not statistically significant for myocardial infarction. Hypertension, myocardial infarction, and cardiovascular diseases had the strongest relationship with the human capital domain, and only stroke had the strongest relationship with the material capital domain. However, social capital showed the weakest relationship with the diseases mentioned above, so this relationship was not significant for any of the diseases. Moreover, since all odds ratios are less than one, all observed relationships are reversed. It can be stated that with the improvement of socioeconomic status (rise in CAPSES score), the chances of suffering from the diseases mentioned above decrease.

**Table 5:** Adjusted Odds Ratios of CAPSES scale and its domains for Predicting CVDs

	Hypertension*		MI**		Stroke***		CVDs****	
	OR(95% CI)	*p*-value	OR(95% CI)	*p*-value	OR(95% CI)	*p*-value	OR(95% CI)	*p*-value
**Material capital**	0.95(0.92–0.98)	< 0.001	0.96 (0.92–1.02)	0.18	0.76(0.68–0.84)	< 0.001	0.95(0.92–0.97)	< 0.001
**Human capital**	0.88 (0.86–0.91)	< 0.001	0.94(0.90 -0.99)	0.017	0.82(0.74–0.90)	< 0.001	0.89(0.87–0.91)	< 0.001
**Social capital**	1.03(0.98–1.09)	0.21	0.92 (0.83–1.02)	0.095	1.08 (0.88–1.32)	0.47	1.002 (0.96–1.05)	0.92
**CAPSES**	0.92(0.87–0.97)	0.002	0.94 (0.85–1.04)	0.27	0.87 (0.75–0.93)	< 0.001	0.93 (0.89–0.98)	0.007

. * The Odds Ratios have adjusted for age, sex, BMI, and smoking

^**^ The Odds Ratios have adjusted for age, sex, BMI, smoking, hypertension, and diabetes

^***^ The Odds Ratios have adjusted for age, BMI, smoking, hypertension, and diabetes

^*^***The Odds Ratios have adjusted for age, sex, BMI, smoking, and diabetes

## Discussion

In this study, we introduced a combined scale of CAPSES. The CAPSES index is one of the composite measures. Characteristics of composite measures are to incorporate several information domains into a singular quantity. The advantage of composite measures is that they offer potentially sophisticated scalar quantities useful for cross-tabulating coarsened or categorized SES measures by outcome measures. The main disadvantage is that they combine constituent information and thus require a strong theory about properly weighing such information [27].

A combined scale of CAPSES uses material capital, human capital, and social capital as capital dimensions that affect the person's SES. This measure merits a bit more attention because of what it reveals about the nature of SES measurement today [27].

We examined the criterion and construct validity of the combined scale of CAPSES. To determine the validity of the structure of the CAPSES scale, its correlation with some common indicators of socioeconomic status was examined. The present study's findings showed that, in general, the correlation between univariate socioeconomic indicators (including education level, occupation, and household expenditure) and combined indicators (including area deprivation index, wealth index, and CAPSES scale) was significant. Correlation magnitudes of the combined scale of CAPSES compared to other socioeconomic indicators were higher. The highest correlation of CAPSES was with the wealth index (0.78) and then with literacy (0.57). This finding was consistent with the Oaks and Rossi study [18]. However, the average correlation values ​​in the present study were slightly lower than that. These differences can be attributed to using different variables and methods in both studies. Because in our study, associations were adjusted by age and sex, while this adjustment was not done in the Oaks and Russian study [18].

The correlation value for the CAPSES scale was 0.444. Overall, the correlation value obtained for this indicator was higher than univariate socioeconomic indicators and the combined indicators such as wealth index and Townsend score. Therefore, the CAPSES scale seems appropriate and acceptable structural validity.

Criterion validity was the second aspect of validity that was addressed in this study. The criterion validity was assessed to identify the relationship between the CAPSES scale and some common health outcomes (including the presence or absence of general health, the general health questionnaire (GHQ), and the presence of chronic disease).

The results of the study showed that the composite scale of CAPSES showed a stronger relationship with both general health status and the mean score of the General Health Questionnaire (GHQ) than other socioeconomic indicators, so a unit increase in the Caps scale could increase the chances of being healthy. The general health questionnaire increased by about 17.5%, and for general health, by about 43%. These results were consistent with a study by Oaks and Rossi [18].

In this study, the combined indicator of CAPSES had a significant and inverse relationship with cardiovascular disease, so a unit increase in the scale of CAPSES reduced the odds of cardiovascular disease by about 7.5%. This finding indicates an inverse relationship between socioeconomic status and cardiovascular disease, consistent with most previous studies.

Among the major forms of cardiovascular disease, the CAPSES scale was significantly associated with stroke and hypertension, which was consistent with studies [28–30] but inconsistent with the results of studies [31, 32]. However, the relationship between the CAPSES scale and myocardial infarction was not significant. The most important reason for the discrepancy between the findings in different studies can be the use of prevalent cases instead of incident cases that can dilute these associations. Another reason might be using different markers as a proxy to determine socioeconomic status.

Of the three components of the CAPSES scale, human capital had the strongest relationship with all three types of cardiovascular disease (hypertension, myocardial infarction, and stroke). Using two important education and employment indicators in this component can be the main reason for the strong relationship between human capital and cardiovascular disease. Both have often been strongly associated with these diseases. Some studies confirmed our results study so that lower educational attainment is associated with a higher prevalence of hypertension [33] and with higher CVD incidence and mortality [34–37]. Longitudinal cohort studies and systematic reviews have demonstrated that the lower the occupational position, the higher the prevalence of hypertension [29], and the higher the CVD incidence and mortality [34, 35, 37]. Another cohort study in the USA found that wealth and income were independently associated with higher stroke risk, but only within the 50–64 age groups [11]. However, Azizkhani showed that there was a significant association between social capital dimensions (i.e., social trust, social support, strong relationship, and hypertension (*P* < 0.05) [38]. Also, Palafox et al. demonstrated that in low-income countries, membership in any social organization was associated with a 3% greater likelihood of having hypertension detected and controlled.

In comparison, greater trust in organizations significantly increased the likelihood of detection by 4%. These associations were not observed among participants in high-income countries [39].In another study, Hwang et al. reported that higher social trust was associated with reduced risk of CVD even after adjustment lifestyle behaviors factors [40]. An individual-level cohort study in 2019 presented that self-reported high social participation was associated with a lower risk for CVD mortality [41].

In the present study, among the socioeconomic indicators studied, the level of education and employment (skill level) at the individual level, household expenditure and wealth index at the household level, and the deprivation index at the group level were considered. In general, among these variables, the education level variable showed the strongest relationship with cardiovascular diseases even after controlling confounding factors. The inequality of cardiovascular disease mortality between lower and higher socioeconomic status, as indicated by educational level, occupational class, or income level, is well-known in many industrialized countries [12]. Socioeconomic status (SES) has shown inverse associations with cardiovascular diseases (CVDs) in most industrialized Western countries, such that disadvantaged groups experience higher risks for CVDs. A considerable portion of the association between SES and CVDs has been attributed to the integrated effects of inequalities in health-related habits and accessibility to the healthcare system [42].

### Strengths and limitations

The large sample size is our study's major strength, which can provide more precise estimates by reducing the probability of random error. Moreover, using a random sample and a high response rate were other major strengths of this study. Another strength of this study was its population-based design, while the most previously conducted studies on cardiovascular diseases, especially myocardial infarction and stroke, were hospital-based. The use of well-trained questioners and several levels of supervisors in this study can be from its strengths. In our study, we used CAPSES socioeconomic index. The use of this index is superior because it includes various aspects of the socioeconomic situation, including material capital, human capital, and social capital, and on the other hand, there is a greater correlation between this index and other socioeconomic determining factors.

On the other hand, the study had several limitations. The inherent limitation of cross-sectional studies, namely the inability to prove the temporality of exposure to the outcome, can lead to reverse causality bias in the inferences. Another limitation was the use of prevalent cases instead of incident cases. It can induce selection bias because the assessed patients were only the cases who survived after their stroke or myocardial infarction and did not include all that occurred. Therefore, the findings of this study may be generalized only to this group of patients and not to fatal cases of the disease.

## Conclusion

Our findings showed that the CAPSES index as a composite measure of SES could be applicable and useful for epidemiological and public health research. It indicated good correlations with conventional SES indexes. Empirical results suggest that all SES measures are related to health. Therefore CAPSES can be used besides other SES indicators as a valid and reliable index that can measure social complexities.

## Supplementary Information

**Additional file 1.**

## Data Availability

The datasets from the current study are included in the article.
