# Supplementary material for: Construction and validation of CAPSES scale as a composite indicator of SES for health research: an application to modeling social determinants of cardiovascular diseases
Source: BMC Public Health. 2023 Feb 9;23:293. doi: 10.1186/s12889-023-15206-9 (PMC9909943; doi:10.1186/s12889-023-15206-9)
Supplement: Supplementary file 1 — Additional file 1. [file 12889_2023_15206_MOESM1_ESM.docx]

**Table 1 supplement. The mean of coefficient correlations for each socioeconomic indicator with the other indicators.**

| **Indicator of socioeconomic status** | **Mean of the coefficients correlations** | **The standard deviation of the correlations** | **Rank of correlations** |
| --- | --- | --- | --- |
| **Literacy** | 0.31 | 160. | 4 |
| **Skill level** | 0.34 | 330. | 3 |
| **Expenditure** | 160. | 40.0 | 6 |
| **Wealth Index** | 350. | 250. | 2 |
| **Townsend index** | 240. | 90.0 | 5 |
| **CAPSES** | 0.44 | 0.23 | 1 |
